# Clinical Equivalence of Monoglyde® and Monocryl® Absorbable Poliglecaprone-25 Sutures: A Single-Blind, Randomized Study

**DOI:** 10.7759/cureus.38938

**Published:** 2023-05-12

**Authors:** MPA Sailakshmi, Sukanta Misra, Sathyashree HS, Soukhin Acharya, Ashok K Moharana, Deepak TS

**Affiliations:** 1 Obstetrics and Gynecology, RajaRajeswari Medical College and Hospital, Bengaluru, IND; 2 Obstetrics and Gynecology, Ramakrishna Mission Seva Pratishthan Vivekananda Institute of Medical Sciences, Kolkata, IND; 3 Clinical Affairs, Healthium Medtech, Bengaluru, IND

**Keywords:** wound composite outcome, subcuticular suturing, poliglecaprone-25 suture, pain score, cesarean delivery

## Abstract

Objectives: Poliglecaprone-25 is a synthetic monofilament suture commonly used for post-cesarean subcuticular skin closure. The present study was designed to assess the effect of subcuticular skin closure using Monoglyde® vs. Monocryl® poliglecaprone-25 absorbable sutures on the risk of wound composite outcomes in the first 30 days post-partum [surgical site infection (SSI), wound dehiscence, hematoma or seroma].

Study Design: This is a prospective, single-blind, randomized (1:1), multicentric, two-arm study performed between September 2020 and December 2021 at two different centers across India. Women (18-40 years) with a singleton pregnancy requiring cesarean delivery were randomized to Monoglyde® (n=62) or Monocryl® (n=62) suture groups. The primary endpoint is the incidence of wound composite outcomes in the first 30 days post-partum (SSI, wound dehiscence, seroma, or hematoma). In addition, the secondary outcomes, incidence of wound composite outcome at all visits (till four months), suture extrusion and loosening, suture removal and evaluation of microbial deposits on sutures (in case not absorbed or infection), operative time, intraoperative suture handling, postoperative pain, return to normal day-to-day activities, modified Hollander cosmesis score, subject satisfaction score, and adverse events were noted.

Results: Non-significant difference between the groups regarding demographic characteristics and primary endpoint; the incidence of wound composite outcome was observed. Moreover, no significant difference in suture extrusion and loosening, suture removal and evaluation of microbial deposits on sutures, operative time, intraoperative suture handling, pain, return to normal day-to-day activities, modified Hollander cosmesis, and subject satisfaction score were registered between the groups.

Conclusions: This study establishes the clinical equivalence of Monoglyde® and Monocryl® poliglecaprone-25 sutures, and both sutures can be used for subcuticular skin closure following cesarean delivery with minimal risk for wound complications.

## Introduction

Cesarean section (CS) is one of the most common surgical procedures, with 6-27.2% rates in developed countries [1]. It reduces maternal and neonatal mortality and morbidity during an emergency [2]. Available data from 150 countries showed that CS accounts for 18.6% of all births [1,3]. Skin closure post-surgery is imperative for wound healing and the mother's recovery. Skin acts as a multifunctional barrier that protects against a wide range of hazardous agents and maintains homeostasis and thermoregulation of the body [4]. Failure of the healing process disrupts the wound, leading to wound issues such as seroma, hematoma, surgical site infections (SSI), etc. Wound issues further result in poor cosmesis, reduced quality of life, protracted hospital stays, and increased economic burden on the patient [5].

The process of wound healing is affected by the structural properties and coating of the suture. Using ideal suture material for subcuticular skin closure not only aids in restoring skin functions but also provides cosmesis [6]. Subcuticular suturing is performed just beneath the epidermal layer and is preferred for post-cesarean skin approximation as it prevents foreign material from passing through [7]. Subcuticular skin suturing following CS has a reduced incidence of wound issues and aesthetically pleasing outcomes [8-10]. Poliglecaprone-25 is an absorbable suture, completely absorbed within 91-119 days of application, with minimal inflammatory reaction [11]. Although existing studies compared poliglecaprone-25 with polyglactin-910 [9] or nylon sutures [11] for subcuticular closure following CS, studies comparing two brands of the same Poliglecaprone-25 material are scarce. Therefore, this study was designed to compare subcuticular skin closure with Monoglyde® or Monocryl® absorbable poliglecaprone-25 sutures for wound composite outcomes (SSI, wound dehiscence, seroma, or hematoma).

## Materials and methods

Study design

This is a prospective, single-blind, randomized (1:1), multicentric, two-arm study. The primary objective was to compare subcuticular skin closure with Monoglyde® or Monocryl® suture for wound composite outcomes in the first 30 days post-partum (SSI, wound dehiscence, seroma, or hematoma). The secondary objectives were to compare the resultant scar and cosmetic effects, overall intraoperative handling, tissue reaction, other adverse events, bacterial accumulation/growth, time taken to resume normal activities, and the post-operative subject satisfaction score in both groups.

Ethics

Institutional Ethics Committee approvals were obtained from both the participating sites. The study includes ethical principles that originated in the Declaration of Helsinki and were conducted by guidelines of MDR (EU) 2017/745, EN ISO 14155:2020, ICH-GCP E6 R2, and Indian MDR rules 2017. The study is registered in the Clinical Trials Registry of India (CTRI/2020/06/025935; Registered on 17/06/2020).

Study participants

Eighteen to 40 years old Primiparous or multiparous women with a singleton pregnancy, good systemic and mental health, and surgical wound classification class I as per Centers for Disease Control and Prevention (CDC), who required CS, were consented and included in the study. 

Women with hemoglobin <7g/dL, body mass index (BMI) >27 Kg/m2, urogenital tract infection within 2 weeks of CS, history of similar surgical procedure (suprapubic transverse scar, Pfannenstiel incision, laparoscopic procedure), bleeding disorders or allergy to the suture materials, and a surgical plan for vertical skin incision were excluded. Women who used an experimental medical device or drug within three months of becoming pregnant or already participating in some other trial and were unlikely to complete the scheduled follow-up visit or comply with the study procedures were also excluded.

Study setting

This study was conducted at two sites in India: (i) Ramakrishna Mission Seva Pratisthan Vivekananda Institute of Medical Sciences, West Bengal, and (ii) Rajarajeswari Medical College and Hospital, Karnataka.

Intervention

The two interventions of this study, Monoglyde® (Healthium) and Monocryl® (Ethicon), are sterile, absorbable, synthetic monofilament, poliglecaprone-25 surgical sutures, which are intended for approximation of tissues.

Randomization and blinding

Computer-generated randomization numbers were used to randomly allocate the study participants to Monoglyde® (n=64) or Monocryl® (n=65) suture group. Subjects were unaware of the intervention status.

Study procedure

On the day of surgery (Visit 1), all routine procedures were followed pre-, peri-, and post-operatively, according to the standard institutional protocol. After completion of the abdominal procedure, either Monoglyde® or Monocryl® suture was used to approximate the abdominal skin. Post-operative follow-up was conducted on Day 3, Day 4-7, Month one, and Month four.

Baseline demographics and characteristics

Age, height, weight, ethnicity, vital signs, parity, gravida, gestation period, fetal presentation, indication for CS, and medical/surgical history were documented.

Study outcomes

Primary Endpoint

The primary endpoint, wound composite outcome: SSI (according to CDC guidelines, SSI occurring within 30 days or up to 90 days after the surgery is superficial or deep SSI, respectively), wound dehiscence (skin separation of ≥1 cm), hematoma or seroma (collection of blood or serous fluid surrounding the wound) within the first 30 days post-partum was assessed.

Secondary Endpoint

The secondary endpoints, the incidence of SSI, wound dehiscence, hematoma, or seroma at all visits, suture extrusion and loosening, suture removal and microbial deposits on sutures (in case not absorbed or infection), operative time (skin incision to closure), intraoperative handling, pain, return to day-to-day activities, modified Hollander cosmesis score, subject satisfaction score, and adverse events were evaluated.

Intraoperative suture handling characteristics viz. first-throw knot holding, ease of passage through tissue, knot security, knot tie-down, stretch capacity, suture fraying, and memory of both sutures were assessed on a five-point scale as follows: 5 excellent, 4 very good, 3 good, 2 fair, and 1 poor. On the day of delivery (after recovery from anesthesia) and during all follow-up visits visual analog scale (VAS) was used to record pain, where 0-4 is no pain, 5-44 mild pain, 45-74 moderate pain, and 75-100 severe pain.

The cosmesis score was evaluated using the modified Hollander Scale. It has 6 clinical variables, edge inversion, step-off borders, excess inflammation, wound margin separation, contour irregularities, and good overall appearance, marked as satisfactory (0) or unsatisfactory (1). The subjects were also asked to grade the scar's general appearance, location, and comfort, using the subject satisfaction score (Likert scale of 1-10, with 10 indicating very satisfied and 1 indicating very unsatisfied) [12].

Other standard details about antibiotic and thrombosis prophylaxis, number and size of sutures, perioperative complications, suture-related challenges, the outcome of surgery, infant birth weight, number of analgesics, and other medications prescribed during the study period were also captured.

Sample size

As reported by a previous study, the cumulative proportion of overall wound complications of 8.3% with subcuticular skin closure was considered in the Monocryl® arm [9]. The anticipated cumulative proportion of the wound complications in the Monoglyde® arm was assumed as 8.8%. With 5% Type I error, 80% power, and a margin of non-inferiority of 15%, the sample size was calculated as 55 in each group giving a total sample requirement of 110. Further, keeping in view post-randomization exclusion and dropout of 20%, the required sample size was finalized to 66 (33 in each arm) per center. After following exclusion criteria, 129 subjects were randomized into (n=64) Monoglyde® and (n=65) Monocryl® groups.

Statistical analysis

The SPSS, Chicago, Illinois, USA (SPSS version 28.0) was used for the study analysis, based on the Per-Protocol(PP) analysis set that includes subjects with complete primary endpoint data for the entire four months' duration. Mean±SD was used to express all continuous variables; for normally distributed data, the t-test was used, and for distribution-free data, the Mann-Whitney U test was used. Fisher's Exact or Chi-square test was used for qualitative variables. Statistical significance was considered when a p-value was <0.05.

## Results

**Figure 1:**
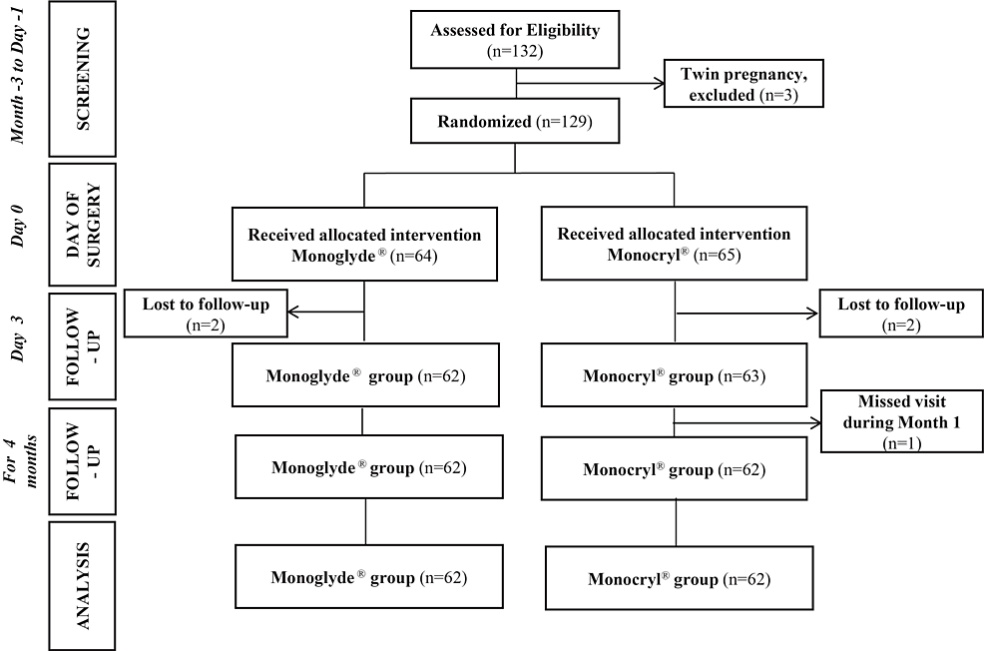
CONSORT flow diagram

Between September 2020 and August 2021, a total of 132 women were screened, and in December 2021, a follow-up of the last subject was completed. The PP set consisted of 124 subjects (excluded, n=3; lost to follow-up, n=4; missed visit, n=1) who received the allocated intervention (Monoglyde®, n=62 and Monocryl®, n=62) and completed the study (Figure 1).

Baseline demographics and other relevant characteristics analysis

All study participants were Indians and had no smoking or alcohol consumption history. Baseline demographics (except weight and height) and other relevant characteristics were comparable (Table 1).

**Table 1:** Baseline demographics and characteristics *p<0.05; / Surgicalcipantsionsween the groups dy inings; CS: Cesarean Section; BMI: Body Mass Index; SD: Standard Deviation

Characteristics	Monoglyde^®^ (n=62)	Monocryl^®^ (n=62)	p value
Demographics			
Age (years), Mean±SD	27.23±5.29	27.51±4.76	0.75
Weight (kg), Mean±SD	63.19±6.36	60.20±5.17	0.01*
Height (cm), Mean±SD	158.00±5.75	155.62±4.59	0.03*
BMI (kg/m^2^), Mean±SD	25.27±1.35	24.85±1.64	0.12
Vital signs			
Pulse rate (beats/minute),Mean±SD	83.87±6.14	83.50±6.35	0.74
Respiratory rate (breaths/minute), Mean±SD	17.45±1.49	17.40±1.67	0.87
Systolic blood pressure (mmHg), Mean±SD	118.06±12.36	117.48±8.25	0.76
Diastolic blood pressure (mmHg), Mean±SD	75.56±6.44	76.32±7.13	0.54
Medical/ Surgical history, n (%)	30 (48.39)	28 (45.16)	0.86
Obstetrics			
Gestation period (weeks), Mean±SD	37.89±1.64	37.65±2.12	0.99
Parity, n (%)			
0	53 (85.48)	56 (90.32)	0.68
1	8 (12.90)	5 (8.06)	
2	1 (1.61)	0	
≥3	0	1 (1.61)	
Gravida, n (%)			
1	43 (69.35)	45 (72.58)	0.78
2	15 (24.19)	12 (19.35)	
≥3	4 (6.45)	5 (8.06)	
Fetal presentation, n (%)			
Breech	3 (4.84)	5 (8.06)	0.98
Cephalic	59 (95.16)	57 (91.94)	
Indication for CS, n (%)			
Fetal distress	33 (53.22)	24 (38.70)	0.15
Intrauterine growth retardation	16 (25.80)	17 (27.42)	
Uncontrolled/ Gestational diabetes mellitus	1 (1.61)	7 (11.29)	
Gestational hypertension	2 (3.22)	4 (6.45)	
Maternal request	10 (16.13)	9 (14.52)	
Intrahepatic cholestasis of pregnancy	0	1 (1.61)	

Although a statistically significant p<0.05 was recorded in weight and height, the BMI (derived variable from weight and height) was non-significant between the studied arms. Hence, the population was not considered heterogeneous, and no subgroup analysis was performed.

Primary endpoint analysis

No incidence of superficial SSI was reported during day 3 or days 4-7. However, one subject in the Monoglyde® group had superficial SSI by the end of the first month. The subject was readmitted to the hospital; pus was drained, re-suturing was done with two stitches at the site of infection, and was treated with intravenous administration of antibiotics for 5 days. The SSI was not related to the device, and the subject continued the study. The finding showed a non-significant difference (p=0.32) between the groups. Furthermore, no wound dehiscence, hematoma, or seroma incidence was reported until the month 1 follow-up.

Secondary endpoint analysis

Intraoperative Profile

**Figure 2:**
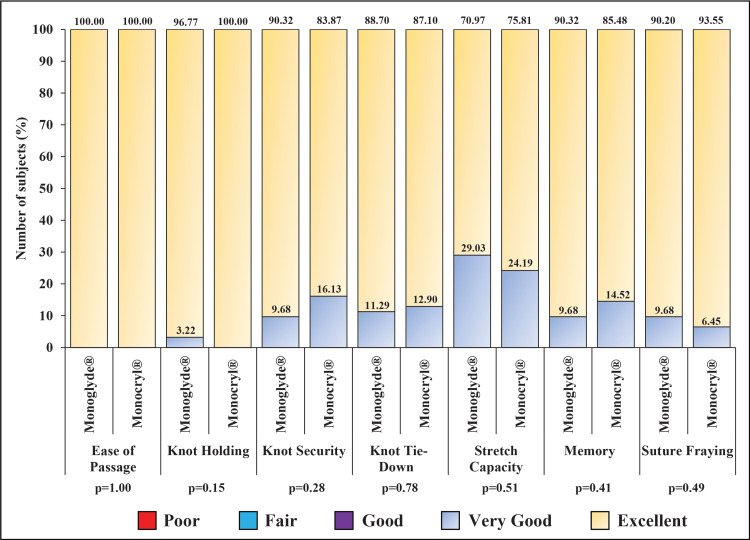
Intraoperative suture handling characteristics of the subjects assigned to Monoglyde® (n=62) and Monocryl® (n=62) groups Percentage scores for “Excellent”, and “Very good” is shown in one bar for different suture handling characteristics. None of the characteristics had “Good” “Fair” or “Poor” score

Spinal anesthesia, antibiotic prophylaxis, along with one suture of 3-0 size was used in all participants. Comparable results for all the suture handling characteristics with "excellent" and "very good" grades and no "good", "fair," or "poor" scores were found between the groups (Figure 2).

Perioperative blood transfusion was required due to atonic post-partum hemorrhage in one subject of the Monoglyde® group, marked as a perioperative complication, and showed no significant difference (p=0.32) with the Monocryl® group. The Other intraoperative characteristics are presented in Table 2. Good outcomes of surgery and no suture-related challenges were noted in both groups.

**Table 2:** Intraoperative and Post-operative profile *p<0.05, SD: Standard Deviation

Characteristics	Monoglyde^®^ (n=62)	Monocryl^®^ (n=62)	p value
Intraoperative profile			
Thrombosis prophylaxis n(%)	18 (29.03)	17 (27.41)	0.84
Total operative time (minutes), Mean±SD	69.53±32.15	67.97±22.67	0.76
Closure of subcutaneous tissue, n (%)	32 (51.61)	32 (51.61)	1.00
Post-operative profile			
Birth weight of infant, Mean±SD	2.80±0.63	2.78±0.49	0.083
Time of onset of pain after surgery (minutes), Mean±SD	70.71±44.03	57.29±28.99	0.047*
Number of analgesics prescribed, Mean±SD			
Day 0	1.71±0.46	1.68±0.47	0.70
Day 3	0.94±0.31	0.94±0.25	1.00
Day 4-7	0.68±0.47	0.69±0.47	0.85
Month 1	0.05±0.22	0	0.08
Month 4	0	0	-
Length of hospital stay (days), Mean±SD	5.35±1.94	5.23±2.08	0.72
Time to resume normal activities (days), Mean±SD	18.11±6.45	18.79±7.02	0.58

Post-operative Profile

**Figure 3:**
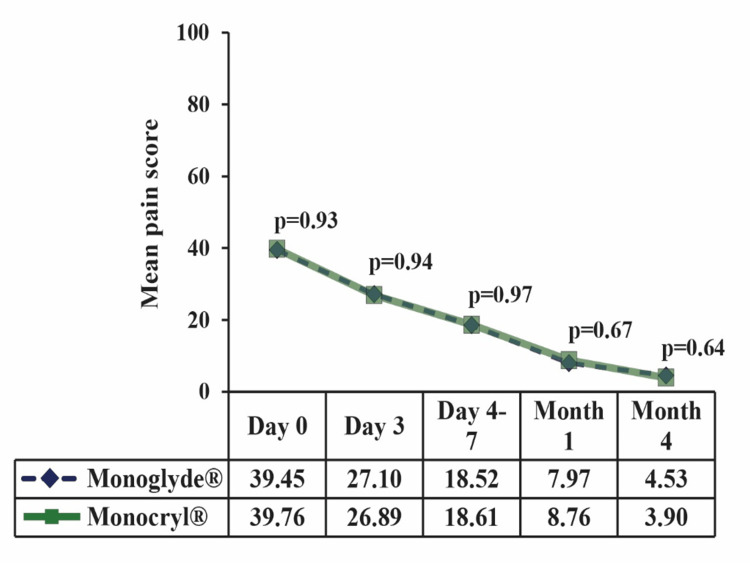
Mean pain score

**Figure 4:**
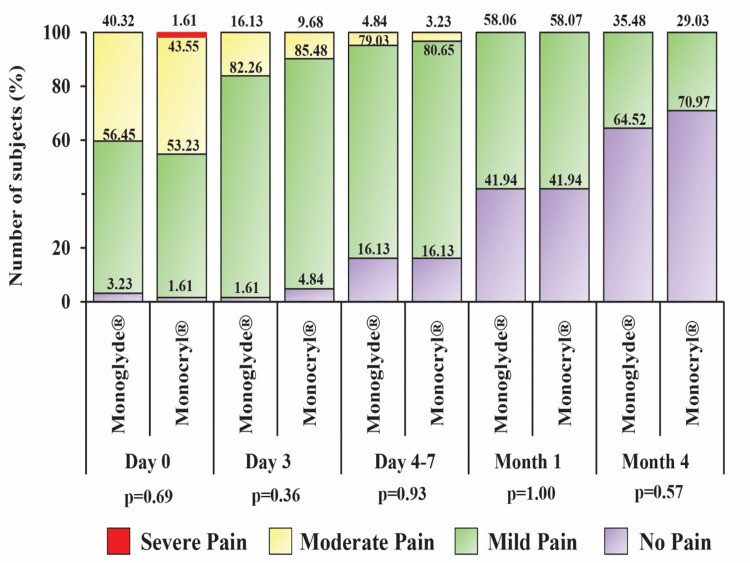
Frequency of subjects with grades of pain (mild, moderate and severe)

The post-operative profile of the subjects is summarized in Table 2. There was no incidence of SSI, wound dehiscence, hematoma, or seroma at the last follow-up (four months). Additionally, no significant difference in the incidence of suture extrusion and loosening, suture removal, and evaluation of microbial deposits on sutures was observed in any subjects. The intensity of pain was comparable between the groups (Figure 3). The grade of pain was also comparable between the groups (Figure 4).

**Figure 5:**
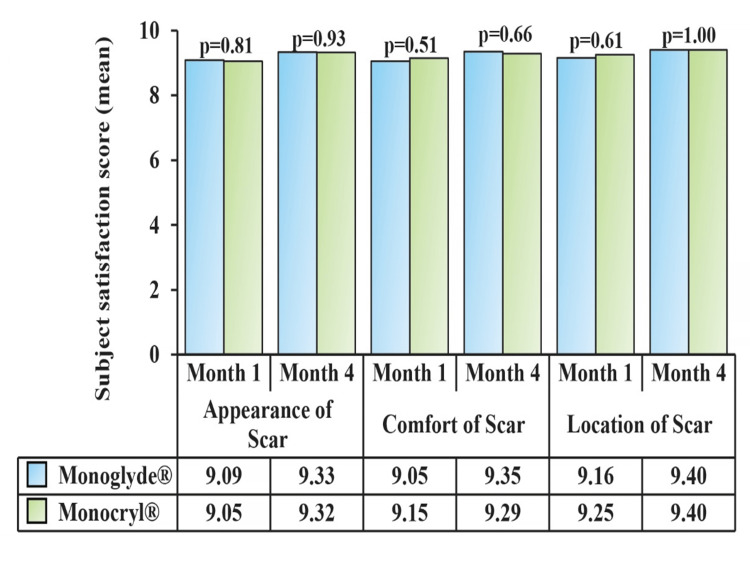
General appearance, location and comfort of the scar were graded using the Likert scale

The one subject in the Monoglyde® group with superficial SSI was readmitted to the hospital and continued the study after recovery. A total cosmetic score of 0 (satisfactory) was recorded in all subjects, and the subject satisfaction score was comparable, with no statistical differences between the groups (Figure 5). The prescribed/concomitant medications during the study period are presented in Table 3.

**Table 3:** Concomitant or prescribed medications

Medications	Monoglyde^®^ (n=62)	Monocryl^®^ (n=62)
Analgesics, n (%)		
Paracetamol	33 (53.23)	33 (53.23)
Diclofenac	33 (53.23)	38 (61.29)
Diclofenac + Serratiopeptidase	22 (35.48)	26 (41.94)
Antibiotics, n (%)		
Cefuroxime	30 (48.39)	28 (45.16)
Metronidazole	60 (96.77)	58 (93.55)
Ceftriaxone	24 (38.71)	23 (37.10)
Cefixime	30 (48.39)	31 (50.09)
Amikacin	32 (51.61)	30 (48.39)
Mupirocin	29 (46.77)	29 (46.77)

Non-serious, device-non-related adverse events, viz. COVID-19 (1.61%), fever (1.61%), cold (3.23%), gastritis (1.61%), and headache (1.61%) in the Monoglyde® group, and COVID-19 (1.61%), fever (3.23%), and headache (3.23%) in Monocryl® group were recorded. Serious adverse event, i.e., hospitalization due to SSI, was reported in one (1.61%) subject of the Monoglyde® group.

## Discussion

Incidence of wound complications after CS ranges between 3-30% and is linked to maternal morbidity and mortality [9,13]. Subcuticular suturing for skin closure following CS provides good cosmetic outcomes with reduced incidence of wound complications [7]. Adequate blood supply at the surgical incision site is crucial for proper wound healing, and subcuticular suturing is reported to provide sufficient blood flow, which may enhance wound healing [14]. An ideal skin closure material delivers a good cosmetic outcome and is associated with low application time, easy handling, and a lower rate of complications [8]. The present study compared subcuticular skin closure with two commonly used brands of absorbable poliglecaprone-25 sutures, Monoglyde® and Monocryl®, for wound composite outcome.

Absorbable poliglecaprone-25 sutures have excellent handling properties as compared to catgut or braided absorbable sutures [15]. Indeed, the result of this study showed "excellent" and "very good" scores for first-throw knot holding, ease of passage through tissue, knot security, knot tie-down, stretch capacity, suture fraying, and memory for both suture brands. Moreover, no suture-related challenges, suture extrusion, loosening, or removal were noted.

In a cohort study, the overall rate of composite wound complication was 16.6%, with infection being the most common. However, the wound composite outcome incidence was lesser with the subcuticular technique using Monocryl 3-0, compared to skin closure using staples [16]. Similarly, the participants of the present study had no incidence of wound dehiscence, seroma, and hematoma after subcuticular skin suturing with absorbable poliglecaprone-25 sutures. The most common hospital-associated infection following CS is reported to be SSI in a previous study, which is responsible for 0.6% of readmission and imposes a substantial burden on the healthcare system, and is also a common cause of morbidity (3-15%) [17]. The incidence of SSI in only one subject of the Monoglyde® group during the Month 1 visit was not related to the studied suture as it was attributed to the patient's poor general condition following intra-operative atonic post-partum hemorrhage during the CS. The subject recovered after treatment and completed the study without further complaints of wound complications.

The aesthetic or cosmetic appearance of scars is pivotal in measuring interventions' effectiveness [18]. Wound healing and recovery of the study participants were evident by the findings of subject satisfaction score along with pain, a requirement of analgesics, and return to day-to-day activity. Scores regarding the scar's general appearance, location, and comfort improved with time. Additionally, the post-operative pain and number of analgesics gradually decreased with each follow-up visit. The time taken to resume regular activity was also similar (~18 days) between the groups. No device-related adverse or serious adverse event was noted in this study.

The results of this study would provide an option to clinicians for selecting Monoglyde® poliglecaprone-25 suture for subcuticular skin closure following CS and all other surgeries indicated for Monocryl® poliglecaprone-25 suture. The study limitation includes (i) the Investigators were not blinded. Hence potential bias might have occurred if they favored one suture over the other, and (ii) as CS is generally considered a clean surgery, the risk of SSI might be lesser.

## Conclusions

This study demonstrated clinical equivalence of Monoglyde® and Monocryl® poliglecaprone-25 sutures as the non-significant difference was observed in the number of participants with surgical site infection, wound dehiscence, hematoma, or seroma within the first 30 days post-partum and at next follow up visit, operative time, the incidence of suture extrusion, loosening, and removal, intraoperative suture handling, pain, evaluation of microbial deposits on sutures with culture, other adverse events and material problems, modified Hollander cosmesis score, return to normal day to day activities, and subject satisfaction score among the groups. Therefore, both sutures can be safely used for subcuticular skin closure following CS with minimal probability of wound complications.

## Appendices

The underlying data related to all the data points (Demographic data, primary and secondary endpoints) are available in the below link:

https://doi.org/10.6084/m9.figshare.20398890.v1

Data are available under the terms of the Creative Commons Attribution 4.0 International license (CC-BY 4.0).
